# Real-world predictors of survival in patients with extensive-stage small-cell lung cancer in Manitoba, Canada: a retrospective cohort study

**DOI:** 10.3389/fonc.2023.1191855

**Published:** 2023-09-18

**Authors:** David E. Dawe, Rebekah Rittberg, Iqra Syed, Mary Kate Shanahan, Daniel Moldaver, Oliver Bucher, Katie Galloway, Kayla Reynolds, James T. Paul, Craig Harlos, Julian O. Kim, Shantanu Banerji

**Affiliations:** ^1^ Department of Internal Medicine, University of Manitoba, Winnipeg, MB, Canada; ^2^ Department of Hematology and Medical Oncology, CancerCare Manitoba, Winnipeg, MB, Canada; ^3^ CancerCare Manitoba Research Institute, CancerCare Manitoba, Winnipeg, MB, Canada; ^4^ AstraZeneca Canada, Mississauga, ON, Canada; ^5^ Department of Epidemiology and Cancer Registry, CancerCare Manitoba, Winnipeg, MB, Canada; ^6^ Department of Cellular & Physiological Sciences, University of British Columbia, Vancouver, BC, Canada; ^7^ Department of Radiology, University of Manitoba, Winnipeg, MB, Canada; ^8^ Department of Radiation Oncology, CancerCare Manitoba, Winnipeg, MB, Canada

**Keywords:** radiotherapy (RT), small cell lung cancer (SCLC), extensive stage (ES), performance status (ECOG-PS), real world, long-term survival, overall survival (OS)

## Abstract

**Background:**

Extensive-stage small-cell lung cancer (ES-SCLC) is an incurable cancer with poor prognosis in which characteristics predictive of long-term survival are debated. The utility of agents such as immune checkpoint inhibitors highlights the importance of identifying key characteristics and treatment strategies that contribute to long-term survival and could help guide therapeutic decisions.

**Objective:**

This real-world analysis examines the characteristics, treatment patterns, and clinical outcomes of patients receiving chemotherapy without immunotherapy for ES-SCLC in Manitoba, Canada.

**Methods:**

A retrospective cohort study assessed patient characteristics, treatment, and survival duration (short: <6 months; medium: 6–24 months; long: >24 months) using the Manitoba Cancer Registry and CancerCare Manitoba records. Eligible patients were aged >18 years with cytologically confirmed ES-SCLC diagnosed between January 1, 2004, and December 31, 2018, and received cytotoxic chemotherapy (CT). The one-, two-, and five-year probabilities of overall survival (OS) were assessed relative to patient, disease, and treatment characteristics using Kaplan-Meier methods and Cox proportional hazards models.

**Results:**

This analysis included 537 patients. Cisplatin was used in 56.1% of patients, 45.6% received thoracic radiotherapy (RT), and few received prophylactic cranial irradiation (PCI). In the overall cohort, one-, two- and five-year OS rates were 26%, 8%, and 3%, respectively. For patients with Eastern Cooperative Oncology Group Performance Status (ECOG PS) 0, OS rates at one, two, and five years were 43%, 17%, and 10%, respectively, vs. 27%, 8%, and 2% for those with ECOG PS 1–2, and 16%, 3%, and 3% for those with ECOG PS 3–4. In long-term survivors, ECOG PS scores were lower and abnormal laboratory test results were less frequent. Overall, 74.4% of long-term survivors received thoracic RT and 53.5% received PCI. Known poor prognostic factors – including brain/liver metastases, high lactate dehydrogenase (LDH), abnormal sodium, and low hemoglobin levels – were less common but still seen in long-term survivors.

**Conclusion:**

Although rare, patients with ES-SCLC may experience long-term survival with CT ± thoracic RT ± PCI. Factors predicting long-term survival include traditional prognostic factors such as ECOG PS, LDH level, and receipt of thoracic RT or PCI. These findings support current treatment algorithms for ES-SCLC and provide baseline survival estimates to assess the real-world impact of adding immune checkpoint inhibitors in the future.

## 1 Introduction

Small-cell lung cancer (SCLC) is an aggressive malignancy characterized by rapid growth and early development of locoregional and distant metastasis (1, 2). SCLC represents an estimated 12% of all lung cancers in Canada (3). It is classified according to disease extent and ability to safely deliver a radical dose of radiotherapy (RT) as either limited stage (LS) or extensive stage (ES). LS-SCLC comprises most patients with 8^th^ edition tumor, node, metastasis (TNM) stage I-IIIB and some with stage IIIC, while ES-SCLC includes the balance of patients with stage IIIC and all patients with stage IV disease (4). Between 60% and 70% of patients with SCLC are diagnosed with ES-SCLC (3, 5, 6), and approximately 95% of ES-SCLC cases are classified as TNM stage IV (7). Prognosis for ES-SCLC has remained poor over the past 20 years (8–10), with a median survival interval of 7–12 months, two-year survival rate of <5%, and five-year survival rate of 1%-2% (2, 6, 11, 12); however, higher survival rates have been observed in some cohorts. Factors associated with poor prognosis of ES-SCLC include poor Eastern Cooperative Oncology Group Performance Status (ECOG PS), multiple metastatic sites, advanced age, elevated lactate dehydrogenase (LDH) level, abnormal serum sodium level, low hemoglobin level, weight loss, poor response to initial treatment, and early relapse (2, 13–18). Conversely, younger age, female sex, good ECOG PS, normal creatinine and LDH levels, and a single metastatic site are favorable prognostic factors in patients with ES-SCLC (14, 16). Patient characteristics and prognostic factors influence patient eligibility for some treatments (1). For example, older patients with ES-SCLC are less likely to receive systemic treatment (1, 19). Some older patients and those with multiple comorbidities may only be considered eligible for best supportive care, which is associated with a worse prognosis (8).

Standard of care in ES-SCLC has traditionally consisted of platinum-based chemotherapy (CT) (cisplatin or carboplatin) plus etoposide; in some populations, etoposide may be replaced with irinotecan (20, 21). Other components of therapy include prophylactic cranial irradiation (PCI) and thoracic RT (8, 9), which may be provided to patients with ES-SCLC who show a tumor response after initial systemic treatment to control local disease and improve overall survival (OS) (10). Platinum-based CT was first demonstrated to be effective in patients with SCLC in 1985 (21, 22) and is associated with better median survival than non-platinum alkylating agents (23, 24). Despite high response rates to first-line CT, almost all patients relapse, require further treatment, and die of progressive disease (2, 25). Relapsed ES-SCLC is associated with low response rates to subsequent therapy and extremely poor prognosis (11).

With CT as the longstanding standard of care for ES-SCLC in Canada, little improvement in prognosis has been observed over decades (26). The recent emergence of immunotherapeutic options has expanded therapy options in this patient population. Immune-checkpoint agents that inhibit programmed death-1 (PD-1) or programmed death ligand 1 (PD-L1) have demonstrated statistically significant improvements in OS in combination with CT vs. CT alone in patients with ES-SCLC (27–29). Health Canada, the United States Food and Drug Administration, and the European Medicines Agency have approved durvalumab (in combination with etoposide and carboplatin or cisplatin) and atezolizumab (in combination with etoposide and carboplatin) as first-line therapy for ES-SCLC (30–35).

With the arrival of new therapeutic strategies, there is an unmet need to understand how baseline patient characteristics influence choice of treatment and clinical outcomes. To date, few real-world studies have comprehensively examined treatment patterns and long-term survival among patients with ES-SCLC in Canada (8, 36, 37). Our group previously evaluated the effect of cisplatin vs. carboplatin on clinical outcomes of a cohort of patients diagnosed with ES-SCLC and LS-SCLC from 2004 to 2013 in Manitoba (37). More patients receiving carboplatin (26.2% of the cohort) had poor ECOG PS, elevated LDH, and ES-SCLC than those receiving cisplatin. Median OS (unadjusted) was 224 vs. 322 days in the carboplatin and cisplatin groups, respectively. We previously performed a separate analysis assessing the impact of hospital admission at the start of CT on outcomes (13). Inpatients had a greater disease burden and poorer ECOG PS than outpatients. ECOG PS was identified on multivariable analysis as an independent predictor of survival.

The current study represents an expansion of this earlier real-world retrospective cohort, with additional data from patients diagnosed up to the year 2018. Immunotherapy first became available for ES SCLC patients in Canada in 2020. The primary objectives were to describe patient characteristics and treatment regimens of patients who received CT for ES-SCLC and to estimate the probability of OS for this cohort to five years from diagnosis. Secondary objectives were to characterize the population of patients with ES-SCLC in Manitoba, to assess the impact of baseline patient and ES-SCLC characteristics and treatment regimens on OS, and to describe the characteristics and treatment regimens of short- (<6 months), medium- (6-24 months), and long-term survivors (>24 months). This study will provide a more complete perspective of the ES-SCLC landscape in Canada.

## 2 Materials and methods

### 2.1 Study design

This is a retrospective, population-based, cohort study of ES-SCLC patients who received CT in the Canadian province of Manitoba, which has a catchment population of approximately 1.4 million universally insured persons with a sole-source, provincially administered, cancer treatment agency (CancerCare Manitoba [CCMB]). This study was approved by the University of Manitoba Health Research Ethics Board (HREB H2015:154 [HS18575]).

### 2.2 Study cohort

Eligible patients 1) were aged >18 years, 2) had cytologically confirmed ES-SCLC (International Classification of Diseases for Oncology [ICD-O] morphology codes 80413, 80423, 80443, 80453), and 3) received cytotoxic CT. Patients who did not receive CT or had non-SCLC or LS-SCLC were excluded from the analysis.

### 2.3 Data source

Data were collected from a previously described study cohort from the Manitoba Cancer Registry (MCR) (13), which is among the oldest cancer registries in North America and is operated by CCMB to collect, classify, and maintain detailed information on all cancer cases in Manitoba. An existing cohort of patients diagnosed between January 1, 2004, and December 31, 2013, was expanded to include patients diagnosed between January 1, 2014, and December 31, 2018. Additional case details for all patients were obtained through a manual review of CCMB electronic medical records. Follow-up data were available until September 30, 2021.

### 2.4 Outcome measures

For the primary and secondary objectives, outcome measures were descriptive of the patient cohort and the treatment regimens they received. Patient characteristics included demographic and clinical information at the time of diagnosis: age, sex, smoking status, stage of disease, laboratory test results, ECOG PS, and location of metastases. Measures of particular interest on laboratory testing included levels of LDH, sodium, and hemoglobin, previously identified as prognostic markers in ES-SCLC (14–17). Our database did not include data on nutritional or inflammatory markers that have shown prognostic value in some studies (38). ECOG PS was obtained based on the description of patient functional status in the initial history and physical examination for patients whenever it was not explicitly stated. Treatment information included regimen received, such as CT (cisplatin or carboplatin) and etoposide, any RT, thoracic RT to lung primary and/or mediastinum (concurrent, consolidative, or palliative), or brain RT (PCI, palliative whole-brain RT). Concurrent RT was given while the patient was receiving CT (planned dose ≥40 Gy). Consolidative RT was given shortly after completion of CT with no evidence of progression and was identified in the physician’s notes as consolidative. Thoracic RT that did not fit into either the concurrent or consolidative categories was categorized as palliative.

Clinical outcomes included treatment response and OS; OS was defined as the time interval (months) from the date of first CT treatment to death, censoring from loss to follow-up, or end of the follow-up period (September 30, 2021). Response to treatment was classified from the clinical records as complete (total resolution of tumor burden), partial (evidence of a decrease in tumor burden without total resolution), stable (no change in tumor burden), progression (increase in tumor burden), or unknown. Patients were classified by survival time: short term (<6 months), medium term (6-24 months), and long term (>24 months). Proportions of patient characteristics, treatment regimens, and patient responses were tabulated and compared by short-, medium-, and long-term survival.

### 2.5 Statistical analysis

Descriptive statistics were used for patient, disease, and treatment characteristics. Frequency (n and %) was determined for each categorical variable of interest, and the median and range were determined for continuous variables. Comparisons were performed using Pearson Chi-square and Fisher exact tests for categorical variables and Kruskal-Wallis tests for continuous and non-normally distributed variables. OS probabilities were estimated for one, two, and five years and were stratified by treatment regimen, age, sex, ECOG PS, smoking status, location of metastases, disease stage at diagnosis, and RT use. Log-rank testing was used to check for statistical significance, with *P*-values ≤0.05 indicative of statistical significance.

Univariable followed by multivariable hazard regression analysis was performed to assess patient, disease, and treatment characteristics associated with OS. The univariable hazard regression associations with *P*-values ≤ 0.2 were entered into the multivariable hazard regression model. To account for the immortality bias associated with having lived long enough to receive lung or brain RT, landmarked survival curves were generated that included only patients who survived ≥6 months. Multivariable Cox regression was performed to determine patient and disease characteristics and treatment regimens associated with prognosis, and *P*-values < 0.05 were considered statistically significant. Splines were used for continuous predictors if they demonstrated a non-linear relationship with the outcome (39). Data analyses were performed in SAS version 9.4 (SAS Institute Inc., Cary, NC, USA) and STATA version 17.0 (StataCorp, College Station, TX, USA).

## 3 Results

### 3.1 Patient demographics

**Figure 1:**
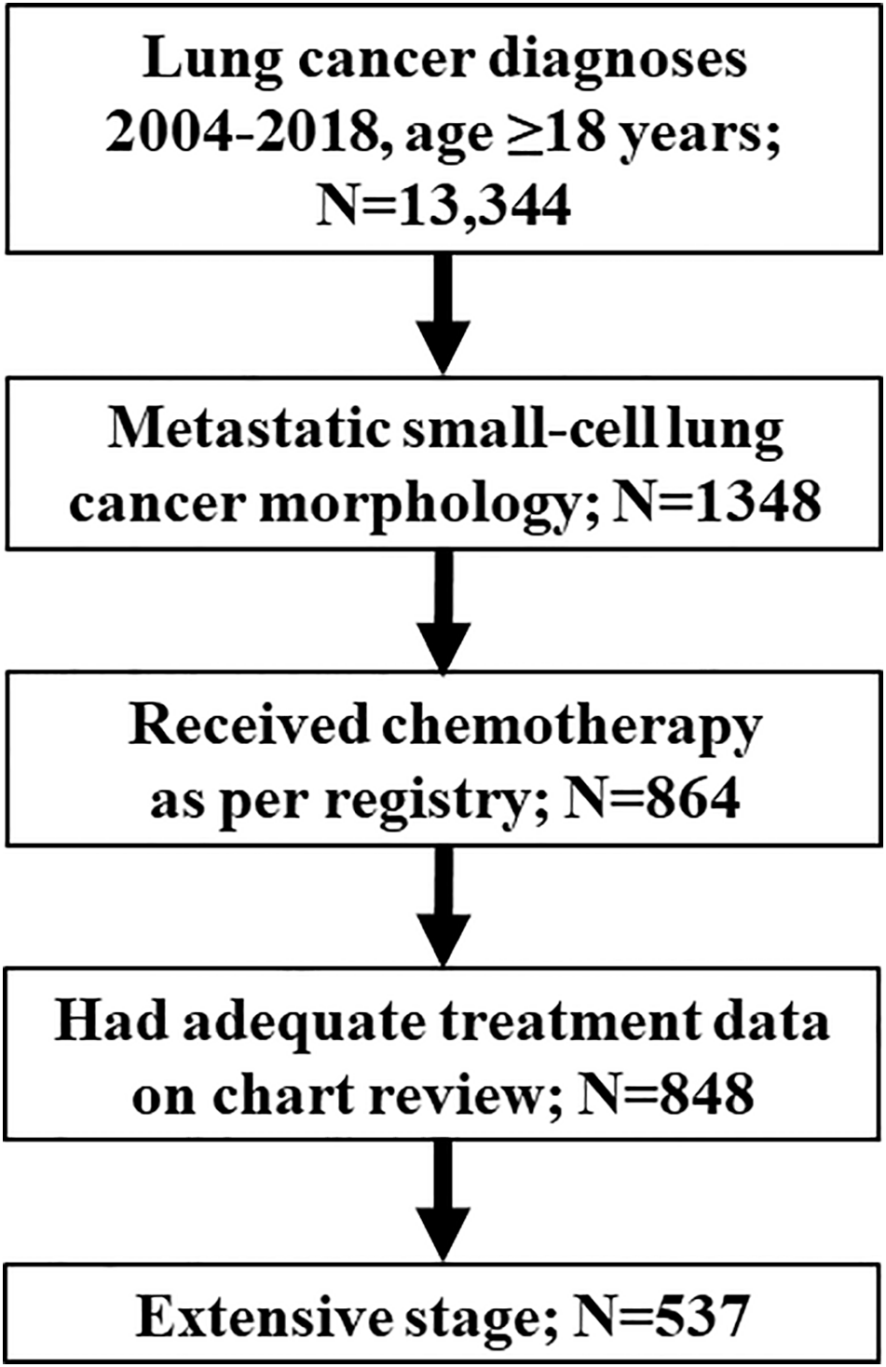
Flowchart of number of eligible cases at each inclusion/exclusion step.

Number of eligible cases at each inclusion step are outlined in **Figure 1**. Patient demographics and disease characteristics for the 537 patients included in the study are summarized by survival subgroup in **Table 1**. The median age of this cohort was 66 (range 38-87) years, with an equal proportion of males and females (49.5% males). The majority of patients (75.6%) had ECOG PS 0-2, and 83.2% of patients had stage IV disease. Long-term survivors were less likely than medium- or short-term survivors to have ECOG PS 3-4, any abnormal laboratory results (LDH, sodium, or hemoglobin levels), or metastases to brain, bone, or liver. Median follow-up time for the entire cohort was 7.7 months. By survivor duration groups, median follow-up times were 3.0 months, 9.8 months, and 40.8 months for short-, medium-, and long-term survivors, respectively.

**Table 1:** Baseline patient and disease characteristics.

Characteristic	Short- term survival	Medium- term survival	Long- term survival	*P*-value^a^
**Patients, n (%)** ^b^	196 (36.5)	298 (55.5)	43 (8.0)	
**Age, years, median (range)**	66 (38-87)	66 (38-87)	61 (47-80)	0.126^c^
**Sex, n (%)**				0.122^d^
**Male**	102 (52.0)	149 (50.0)	15 (34.9)	
** Female**	94 (48.0)	149 (50.0)	28 (65.1)	
**ECOG PS, n (%)**				<0.001
** 0**	7 (3.6)	40 (13.4)	−^e^	
** 1-2**	117 (59.7)	203 (68.1)	28 (65.1)	
** 3-4**	69 (35.2)	54 (18.1)	−^e^	
**Brain metastases, n (%)**				0.116
** Yes**	27 (13.8)	25 (8.4)	−^e^	
**Liver metastases, n (%)**				<0.001
** Yes**	61 (31.1)	61 (20.5)	−^e^	
**Bone metastases, n (%)**				0.001
** Yes**	46 (23.5)	37 (12.4)	−^e^	
**Collaborative stage, n (%)**				0.237
** III**	21 (10.7)	52 (17.5)	10 (23.3)	
** IV**	173 (88.3)	241 (80.9)	33 (76.7)	
**Smoking status, n (%)**				0.732
** Never/ex-smoker/unknown**	133 (67.9)	193 (64.8)	24 (55.8)	
** Current**	63 (32.1)	105 (35.2)	19 (44.2)	
**LDH,****^f^** **n (%)**				<0.001
** Normal**	39 (19.9)	113 (37.9)	28 (65.1)	
** Elevated**	135 (68.9)	171 (57.4)	12 (27.9)	
**Sodium,****^g^** **n (%)**				0.014
** Abnormal**	61 (31.1)	76 (25.5)	7 (16.3)	
**Hemoglobin,****^h^** **n (%)**				0.038
** Low**	101 (51.5)	149 (50.0)	18 (41.9)	

a Fisher exact test P-value.

b unknown data comprise the differences in characteristic subtotals and the group totals.

c Kruskall Wallis test P-value.

d Chi-square P-value.

e patient numbers ≤5 are censored based on requirements from Manitoba Health.

f elevated LDH: >230 U/L.

g abnormal sodium: <135 or >147 mEq/L.

h low hemoglobin: males <140 g/L, females <120 g/L. ECOG PS, Eastern Cooperative Oncology Group Performance Status; LDH, lactate dehydrogenase.

Treatment response outcomes and pattern of treatment regimens are presented by survival subgroup in **Table 2**. Complete/partial response was experienced by 61.3% of patients. More patients received cisplatin than carboplatin (56.1% vs. 43.2%) as first-line therapy, 71.1% of patients underwent RT, and < 15% received PCI. At least four cycles of CT were completed by 97.7% of long-term survivors, 92.6% of medium-term survivors, and 35.2% of short-term survivors, although delayed courses were more frequent (81.4%) with long-term survivors than with medium- or short-term survivors (71.8% and 35.7%, respectively). Thoracic RT was administered to 74.4% of long-term survivors, 53.7% of medium-term survivors, and approximately one-quarter of short-term survivors, respectively. PCI was administered to 53.5% of long-term survivors, 13.8% of medium-term survivors, and a minimal number of short-term survivors.

**Table 2:** Treatment characteristics by survival.

Characteristic	Short- term survival	Medium- term survival	Long- term survival	*P*-value^a^
Patients, n (%)^b^	196 (36.5)	298 (55.5)	43 (8.0)	
Response, n (%)				<0.001
Complete or partial	49 (25.0)	242 (81.2)	38 (88.4)	
Stable	7 (3.6)	18 (6.0)	−^c^	
Progression	34 (17.4)	28 (9.4)	−^c^	
Unknown	106 (54.1)	10 (3.4)	−^c^	
Chemotherapy, n (%)				0.006
Cisplatin	94 (48.0)	180 (60.4)	27 (62.8)	
Carboplatin	99 (50.5)	118 (39.6)	15 (34.9)	
1st chemotherapy setting, n (%)				<0.001
Inpatient	57 (29.1)	42 (14.1)	7 (16.3)	
Outpatient	139 (70.9)	256 (85.9)	36 (83.7)	
Course of therapy, n (%)				
Completed chemotherapy	69 (35.2)	276 (92.6)	42 (97.7)	<0.001
Dose reduction	50 (25.5)	84 (28.2)	12 (27.9)	0.801
Course delayed	70 (35.7)	214 (71.8)	35 (81.4)	<0.001
Lung RT delivery, n (%)				<0.001
None	143 (73.0)	138 (46.3)	11 (25.6)	
Concurrent/consolidative	−^c^	49 (16.4)	20 (46.5)	
Palliative	47 (24.0)	111 (37.3)	12 (27.9)	
PCI received, n (%)				<0.001
Yes	−^c^	41 (13.8)	23 (53.5)	

a Fisher exact test P-value;

b unknown data comprise the differences in characteristic subtotals and the group totals;

c patient numbers ≤5 are censored based on requirements from Manitoba Health. PCI, prophylactic cranial irradiation; RT, radiotherapy.

### 3.2 Survival analysis

**Figure 2:**
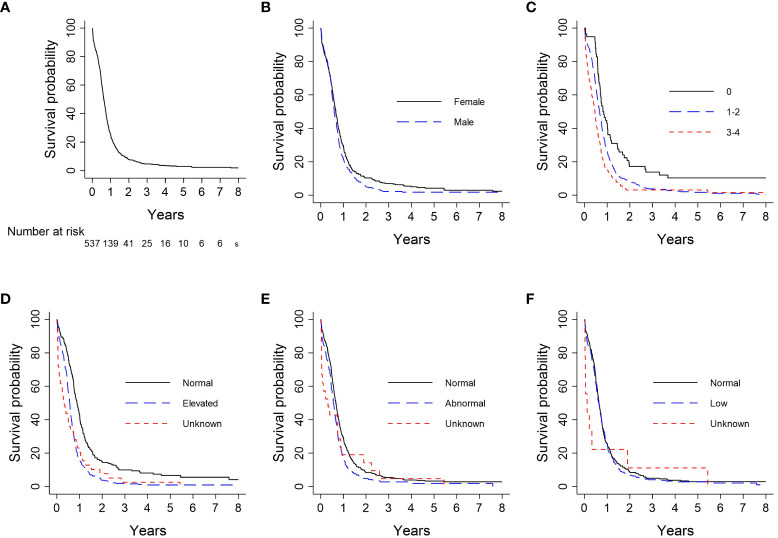
Analysis of OS by patient characteristic: **(A)** Overall cohort (n = 537); **(B)** Sex (n = 537); **(C)** ECOG PS (n = 533); **(D)** LDH (n = 537); **(E)** Serum sodium (n = 536); **(F)** Hemoglobin (n = 537). CI, confidence interval; ECOG PS, Eastern Cooperative Oncology Group Performance Status; Hgb, hemoglobin; LDH, lactate dehydrogenase; OS, overall survival.

**Figure 3:**
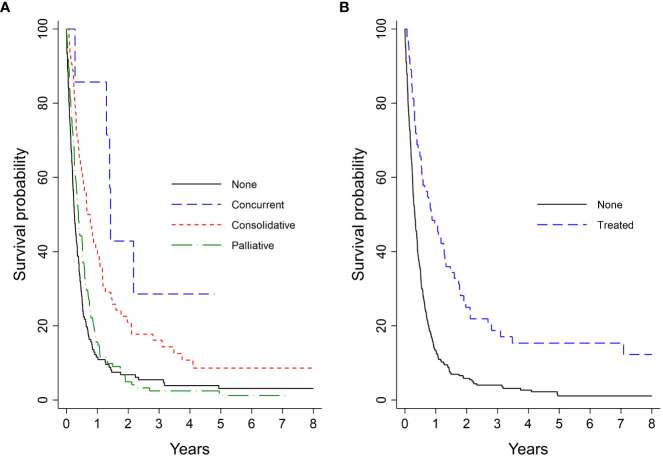
Analysis of extensive-stage patient’s OS by treatment pattern landmarked at 6 months (n = 338): **(A)** Type of lung RT; **(B)** PCI. OS, overall survival; PCI, prophylactic cranial irradiation; RT, radiotherapy.

Kaplan-Meier analysis for survival within the entire cohort demonstrated OS estimates at one, two, and five years of 26%, 8%, and 3%, respectively (**Figure 2A**). Females were more likely than males to survive to two years (10% vs. 5%) and five years (4% vs. 2%; log-rank *P* = 0.05; **Figure 2B**). For patients with ECOG PS 0, the one-, two-, and five-year survival rates were 43%, 17%, and 10%, respectively, vs. 27%, 8%, and 2%, for patients with ECOG PS 1-2, and 16%, 3%, and 3%, for patients with ECOG PS 3-4. Patients with an initial ECOG PS of 0 were significantly more likely to survive to one, two, and five years than patients with an initial ECOG PS ≥ 1 (log-rank *P* < 0.01; **Figure 2C**). Patients with normal LDH and sodium levels at diagnosis had significantly higher survival rates than those with elevated LDH (log-rank *P* < 0.01; **Figure 2D**) and abnormal sodium (log-rank *P* = 0.01; **Figure 2E**), respectively. The survival probability was not significantly different for normal vs. low hemoglobin level (log-rank *P* = 0.53; **Figure 2F**). Median OS and interquartile range (IQR) for concurrent, consolidative, and palliative thoracic RT were 1.9 (IQR 0.8-upper bound missing), 1.1 (IQR 0.9-1.4), and 0.7 (IQR 0.6-0.8) years, respectively. Landmarked OS analysis performed in patients who survived for six months or longer showed a significant difference in OS by type of RT (*P* < 0.01) (**Figure 3A**). Median OS was 1.3 years among patients treated with PCI, and survival rates were higher among PCI recipients than those who did not receive PCI. Landmarked OS analysis at six months supported longer median OS with PCI use (*P* < 0.01; **Figure 3B**).

**Figure 4:**
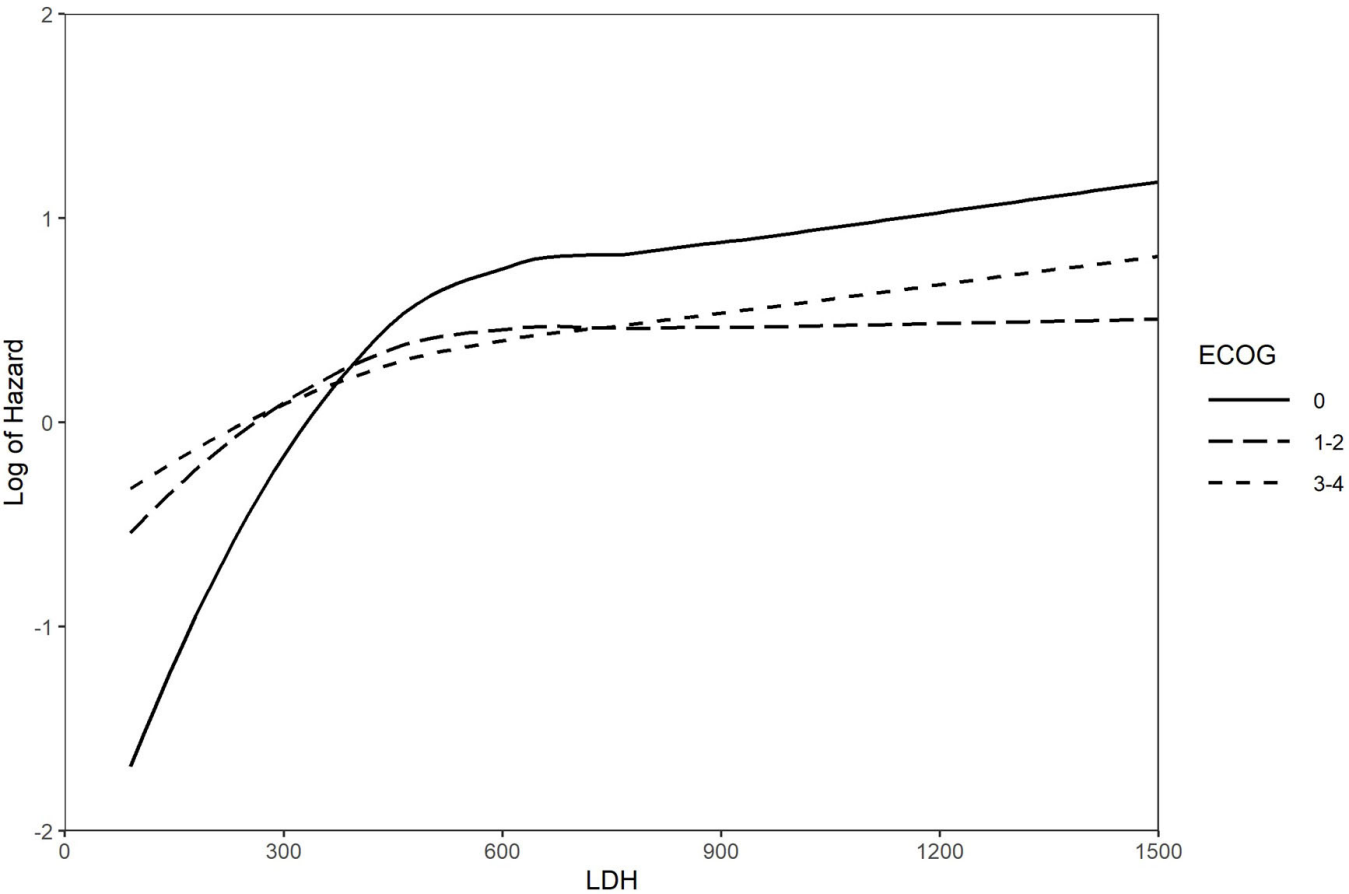
Log of the hazard for the interaction between LDH and ECOG PS Landmarked analysis at 6 months; multivariable models (n = 322). ECOG PS, Eastern Cooperative Oncology Group Performance Status; LDH, lactate dehydrogenase.

On univariable analysis, survival was significantly associated with ECOG PS, CT completion, age at diagnosis, LDH level, PCI, and thoracic RT (**Supplementary Table 1**). On multivariable analysis, use of PCI and lung RT (none, concurrent, consolidative, palliative) as well as completion of CT were independent predictors of improved OS, while poor ECOG PS and increased LDH level was an independent predictor of reduced OS (**Table 3**). A landmarked multivariable Cox analysis examining only patients surviving for six months or longer was performed to account for immortality bias, and it showed a similar pattern of significant associations (**Table 4**). Interaction between LDH and ECOG PS was also found to be significant in this landmarked model (**Figure 4**).

**Table 3:** Multivariable analysis of the full cohort (N = 492).

Variable	Categories	HR	Lower 95% CI	Upper 95% CI	*P*-value	Overall *P*-value
ECOG PS	0	Reference	–	–	–	
	1-2	1.63	1.21	2.21	0.001	
	3-4	1.97	1.39	2.80	<0.001	0.0003
LDH	‘^a^	1.01	1.00	1.01	0.01	
	‘‘^a^	0.89	0.73	1.07	0.22	
	‘‘‘^a^	1.19	0.88	1.60	0.25	<0.0001
Lung RT (original)	None	Reference	–	–	–	
	Concurrent	0.15	0.06	0.42	<0.001	
	Consolidative	0.58	0.42	0.80	0.001	
	Palliative	0.75	0.61	0.92	0.005	<0.0001
PCI	No	Reference	–	–	–	
	Yes	0.39	0.28	0.55	<0.001	<0.0001
Treatment	Cisplatin (I)	Reference	–	–	–	
	Cisplatin (C)	0.46	0.32	0.66	<0.001	
	Carboplatin (I)	1.03	0.72	1.46	0.89	
	Carboplatin (C)	0.46	0.32	0.68	<0.001	<0.0001

a Spline transformations according to knots at the 5^th^ (143), 35^th^ (220), 65^th^ (351), and 95^th^ (1474) percentiles. CI, confidence interval; ECOG PS, Eastern Cooperative Oncology Group Performance Status; HR, hazard ratio; LDH, lactate dehydrogenase; PCI, prophylactic cranial irradiation; RT, radiotherapy; SE, standard error; I, incomplete course; C, complete course.

**Table 4:** Multivariable analysis of the landmarked cohort of patients with ES-SCLC who survived to 6 months (N = 322).

Variable	Categories	HR	Lower 95% CI	Upper 95% CI	*P*-value	Overall *P*-value
ECOG PS	0	Reference	–	–	–	
	1-2	6.10	1.33	27.93	0.02	
	3-4	9.30	1.58	54.88	0.01	0.05
LDH	‘^a^	1.01	1.00	1.02	0.001	
	‘‘^a^	0.98	0.97	1.00	0.008	<0.0001
Lung RT (original)	None	Reference	–	–	–	
	Concurrent	0.20	0.07	0.56	0.002	
	Consolidative	0.62	0.43	0.89	0.01	
	Palliative	0.85	0.65	1.10	0.21	0.002
PCI	No	Reference	–	–	–	
	Yes	0.46	0.33	0.64	<0.001	<0.0001
LDH x ECOG PS	LDH’ * ECOG 1-2	0.99	0.99	1.00	0.07	
	LDH’’ * ECOG 1-2	1.01	1.00	1.02	0.16	
	LDH’ * ECOG 3-4	0.99	0.99	1.00	0.04	
	LDH’’ * ECOG 3-4	1.01	1.00	1.03	0.08	0.07

a Spline transformations according to knots at the 10^th^ (161), 50^th^ (258), and 90^th^ (744) percentiles. CI, confidence interval; ECOG PS, Eastern Cooperative Oncology Group Performance Status; HR, hazard ratio; LDH, lactate dehydrogenase; PCI, prophylactic cranial irradiation; RT, radiotherapy; SE, standard error.

## 4 Discussion

This real-world population-based study describes patient and disease characteristics, patterns of clinical practice prior to availability of immunotherapy, and treatment outcomes in patients with ES-SCLC who received CT as part of their treatment. To our knowledge, this is the most up-to-date population-based study to comprehensively examine treatment patterns and clinical outcomes in ES-SCLC in Canada. Our results are consistent with other studies of long-term survivors with ES-SCLC, indicating that good ECOG PS, good response to CT, and use of thoracic RT and PCI are associated with longer survival in patients with ES-SCLC (2, 11, 40, 41). In particular, our analysis provides additional evidence to the existing literature that ECOG PS is a strong independent predictor of survival in patients with SCLC treated with CT (1, 13). Patients with ECOG PS 0 had a significantly higher median survival (0.82 years) and five-year survival probability (10%) than those with ECOG PS 1-2 (0.67 years; 2%) or 3-4 (0.46 years; 3%). These findings align with the five-year OS in our earlier study using data from part of the current cohort of patients diagnosed prior to December 31, 2013 (ECOG PS 0: 10.7%; 1-2: 3.1%; 3-4: 2.8%) (13). A real-world analysis of 988 patients in China with SCLC showed a significantly longer median OS among patients with ES-SCLC (n = 507) and ECOG PS 0-1 vs. 2-3 (12.0 [95% confidence interval (CI): 11.0-13.0] vs. 9.0 [95% CI 6.9-11.1] months) (42). Another real-world study based in China of 358 patients with ES-SCLC found no significant difference in the median OS between ECOG PS 0-1 and 2-4 (14.5 vs. 9.3 months; hazard ratio [HR] 1.37; *P* = 0.095); however, only 9.8% of their cohort (N = 358) had ECOG PS ≥ 2 (14).

Associations were also identified between long-term survival and other factors, such as LDH, serum sodium, and hemoglobin levels. Outcomes did not differ based on the type of CT received, but survival was superior in patients who completed CT. While there seems to be a global (including Canada) preference for carboplatin over cisplatin (8), cisplatin was first-line therapy in a higher proportion of our patients. Carboplatin- and cisplatin-containing regimens have been found to be similarly efficacious, with no significant differences in response rate, progression-free survival, or OS (43, 44). Nearly all (97.7%) of the long-term survivors in our study completed their CT regimen, despite it including delays in 81.4% of cases. Survival of our cohort – 26%, 8%, and 3% at one, two, and five years, respectively – aligns with findings from other population-based studies in Canada, contributing to the improved generalizability of these data in the aggregate. A retrospective, longitudinal, cohort study in Alberta, Canada used population-level data to describe treatment patterns, demographic and clinical characteristics, and OS of patients with ES-SCLC (2010–2018) (8). Median OS among those receiving first-line CT (46.5% of the total cohort; n = 903) was 7.8 months (95% CI 7.5–8.2) and was 5.7 (95% CI 4.9–6.9) and 3.8 (95% CI 3.0–4.6) months when CT was used as second- and third-line treatment, respectively. Five-year OS was 2.9% (95% CI 1.8–4.5) in the group that received first-line CT. Another real-world study in Alberta included patients with LS-SCLC and ES-SCLC managed at a tertiary cancer center (36). First-line CT was used in 90% of ES-SCLC patients (53% CT alone, 17% with thoracic RT, and 20% with immunotherapy, nonthoracic RT, or metastatic resection), and 20% received PCI. Median OS was 9 months with first-line CT and 13 months among patients treated with a combination of CT and thoracic RT. As in our study, these investigators found PCI to be an independent predictor of improved OS (HR 0.48; 95% CI 0.3-0.7; *P* < 0.01); however, analysis of outcomes related to PCI are likely biased by preferential administration to healthier patients.

OS rates in this study are also consistent with those of CT-only control arms in recent randomized, controlled trials of immune checkpoint inhibitors (ICIs) (27–29). The CASPIAN study of first-line durvalumab plus CT (etoposide plus either carboplatin or cisplatin) reported a one-year OS for the placebo plus CT-control arm of 40% (27). The IMpower133 study of first-line atezolizumab plus CT (carboplatin and etoposide) reported a one-year OS of 38% in the CT-only control arm (28), and the KEYNOTE-604 study of first-line pembrolizumab plus CT (etoposide plus either carboplatin or cisplatin) determined one- and two-year OS in the CT-only control arm of 40% and 11%, respectively (29). As may be expected for a real-world population, our cohort was slightly older (median 66 years), had poorer ECOG PS status, and had a higher proportion of patients with brain metastases than the control arms of the clinical trials. Nevertheless, the subgroup of our cohort with ECOG PS 0 had one-year OS of 43% and two-year OS of 17%.

The CASPIAN and IMpower133 trials through *post hoc* analyses determined that long-term survival was higher among patients receiving an ICI with standard CT as first-line therapy vs. CT alone. In CASPIAN, OS at 18 months was 34% in the group that received durvalumab with CT (cisplatin or carboplatin) plus etoposide vs 25% for CT and etoposide alone (27). In IMpower133, OS at 18 months was 34.0% for atezolizumab with CT (carboplatin) plus etoposide vs 21.0% for CT and etoposide alone (45). OS at 24 and 36 months in CASPIAN extensions were 22.2% and 17.4%, respectively, for the durvalumab plus CT-etoposide group vs 14.4% and 5.8%, respectively, for CT-etoposide alone (46, 47). Similar to our findings, long-term survivors in CASPIAN (i.e., those still alive after the data cutoff; median follow-up 39.4 months) were more likely than short- or medium-term survivors to have favorable prognostic characteristics such as ECOG PS 0 and absence of brain or liver metastases (48). In the long-term survivor subgroup of the IMpower133 trial (median follow-up 22.9 months), a between-treatment difference of > 5% was found for characteristics including age ≥65 years, sex, ECOG PS 0, LDH level, and presence of brain metastases (49). The control arms of these recent trials highlight the continued poor prognosis of patients receiving CT alone for ES-SCLC. Immunotherapy is changing the treatment algorithm in ES-SCLC, suggesting that platinum-based doublet CT combined with an ICI is becoming the new standard of care (50). The advent of new therapeutic options in SCLC emphasizes the need to target prognostic factors and individualize treatment decision-making (2). The similarity of results seen between real world populations and trial control arms suggests that the improved survival seen in trials of CT plus immunotherapy could bear out in future real-world assessments. Additional research is needed to elucidate factors that influence the survival of patients with SCLC treated with CT in combination with immunotherapy. Questions also remain regarding the safety and impact of consolidative thoracic RT in patients receiving CT + ICI regimens, since the landmark trials of CT + ICI did not include consolidative thoracic RT. Based on early phase and cohort data, a recent Canadian guideline suggests considering some patients receiving CT + ICI for consolidative thoracic RT (51). It remains unclear whether the prognostic benefit we saw in our cohort with no ICI will translate to patients who receive CT + ICI.

### 4.1 Study limitations

While observational and retrospective studies are prone to selection bias, our use of a population-based registry sample of all eligible CT treated patients throughout Manitoba was intended to minimize that risk. However, the study cohort was limited to patients who received CT and, therefore, those who survived long enough to receive treatment, which introduces selection bias. This is a particular issue for treatments given almost exclusively after CT, such as thoracic RT or PCI. To adjust for immortality bias, landmarked analyses were performed. This being a retrospective study, some of the data on ECOG PS were derived from patient description in the medical chart instead of explicitly recorded values, leading to a risk of misclassification. Of the patient records analyzed, there were missing laboratory values (2%-7% of patients for the laboratory measures of interest) and limited information on brain, liver, and bone metastases for patients diagnosed prior to 2010. Our cohort also does not include any patients who received chemotherapy plus ICIs, since these regimens were not available in Manitoba during the studied period. The absence of patients receiving these regimens means that our results should not be extrapolated to patients receiving chemotherapy plus an ICI.

## 5 Conclusions

Our study provides supporting evidence that long-term survival with ES-SCLC occurs. Findings from this real-world data further support the association between long-term survival and known prognostic factors such as ECOG PS, laboratory values, and receipt of RT in addition to CT. With the recent introduction of immunotherapy into the routine clinical management of SCLC, future real-world evidence can characterize the long-term responders to ICIs.

## Data availability statement

The datasets presented in this article are not readily available because the data used in this analysis are owned by the government of Manitoba. The authors were given permission to use the data to conduct the analysis. However, they do not have permission to share the data. The authors did not have special access privileges and interested researchers would be able to access the data in the same manner as the authors. Requests to access the datasets should be directed to the Provincial Health Research Privacy Committee, Research Manitoba, A201 Chown Building, 753 McDermot Avenue, Winnipeg MB, R3E 0T6 (email: phrpc@researchmb.ca) and CancerCare Manitoba. Instructions can be found at: https://www.rithim.ca/phrpc-submission-information and https://www.cancercare.mb.ca/Research/research-impact-commitee.

## Ethics statement

The studies involving humans were approved by University of Manitoba Health Research Ethics Board. The studies were conducted in accordance with the local legislation and institutional requirements. The ethics committee/institutional review board waived the requirement of written informed consent for participation from the participants or the participants’ legal guardians/next of kin because almost all patients had died of their disease and no identifiable data would be reported. This study used cancer registry data and retrospective chart review.

## Author contributions

DD: trial design, data curation, formal analysis, visualization, writing – review and editing. RR: data acquisition, formal analysis, visualization, writing – review and editing. IS: writing – review and editing. MS: writing – review and editing. DM: writing – review and editing. OB: data curation, formal analysis, writing – review and editing. KG: data curation, formal analysis, writing – review and editing. KR: data acquisition, writing – review and editing. JP: writing – review and editing. CH: writing – review and editing. JK: writing – review and editing. SB: writing – review and editing. All authors contributed to the article and approved the submitted version.
