# Supplementary material for: Real-world predictors of survival in patients with extensive-stage small-cell lung cancer in Manitoba, Canada: a retrospective cohort study
Source: Front Oncol. 2023 Sep 18;13:1191855. doi: 10.3389/fonc.2023.1191855 (PMC10545857; doi:10.3389/fonc.2023.1191855)
Supplement: Supplementary file 1 [file Table_1.docx]

**Supplementary Table 1: Univariable hazard regression analysis of full cohort (N = 533)**

| Variable | Categories | Hazard ratio | Standard error | Lower 95% CI | Upper 95% CI | *P*-value | Overall *P*-value |
| --- | --- | --- | --- | --- | --- | --- | --- |
| ECOG | 0 | Reference | - | - | - | - |  |
|  | 1‒2 | 1.66 | 0.25 | 1.24 | 2.23 | 0.001 |  |
|  | 3‒4 | 2.35 | 0.39 | 1.69 | 3.26 | <0.001 | <0.0001 |
| Treatment | Cisplatin (I) | Reference | - | - | - | - |  |
|  | Cisplatin (C) | 0.38 | 0.07 | 0.27 | 0.54 | <0.001 |  |
|  | Carboplatin (I) | 1.12 | 0.19 | 0.81 | 1.56 | 0.49 |  |
|  | Carboplatin (C) | 0.42 | 0.08 | 0.29 | 0.60 | <0.001 | <0.0001 |
| Sex | Female | Reference | - | - | - | - |  |
|  | Male | 1.18 | 0.10 | 0.99 | 1.40 | 0.07 | 0.07 |
| Age at diagnosis | Continuous | 1.01 | 0.005 | 1.00 | 1.02 | 0.02 | 0.02 |
| LDH | Continuous | 1.00 | 0.00008 | 1.00 | 1.00 | <0.001 | <0.0001 |
| Na | Continuous | 0.99 | 0.008 | 0.97 | 1.00 | 0.07 | 0.07 |
| Hgb | Continuous | 1.00 | 0.002 | 0.99 | 1.00 | 0.39 | 0.39 |
| Lung RT | No | Reference | - | - | - | - |  |
|  | Yes | 0.53 | 0.05 | 0.44 | 0.63 | <0.001 | <0.0001 |
| Lung RT (original) | None |  |  |  |  |  |  |
|  | Concurrent | 0.19 | 0.09 | 0.08 | 0.46 | <0.001 |  |
|  | Consolidative | 0.35 | 0.05 | 0.27 | 0.47 | <0.001 |  |
|  | Palliative | 0.68 | 0.07 | 0.56 | 0.83 | <0.001 | <0.0001 |
| PCI | No | Reference | - | - | - | - |  |
|  | Yes | 0.34 | 0.05 | 0.26 | 0.45 | <0.001 | <0.0001 |

ECOG PS, Eastern Cooperative Oncology Group Performance Status; CI, confidence interval; LDH, lactate dehydrogenase; Na, sodium; Hgb, hemoglobin; PCI, prophylactic cranial irradiation; RT, radiotherapy.
